# Determining diagnosis of scaphoid healing: Comparison of cone beam CT and X-ray after six weeks of immobilization

**DOI:** 10.1016/j.ejro.2020.100251

**Published:** 2020-09-02

**Authors:** Lucia Calisto Farracho, Berenice Moutinot, Angeliki Neroladaki, Marion Hamard, Karel Gorican, Pierre Alexandre Poletti, Jean Yves Beaulieu, Cindy Bouvet, Sana Boudabbous

**Affiliations:** a Division of Radiology, Department of Diagnosis, Geneva University Hospitals, Rue Gabrielle-Perret-Gentil 4, 1211, Geneva 14, Switzerland; b Hand Surgery Unit, Orthopedic and Traumatology Division, Surgery Department, HUG, Switzerland

**Keywords:** Scaphoid healing, CBCT, X-rays, Immobilisation

## Abstract

**Purpose:**

To assess the accuracy and reliability of using cone beam computed tomography (CBCT) compared to X-ray six weeks after injury for predicting scaphoid union.

**Materials and methods:**

Overall; 52 patients with scaphoid fractures between April 2018 and March 2019 were prospectively included in this study. The mean age was 34.52 (13–88) years, and the gender ratio male/female 43/9. Of the fractures, 26 had occurred on the right side, and 26 on the left side. In total, 28 % of patients were manual workers. All patients underwent X-ray and CBCT six weeks after injury. Four readers, two radiologists, and two hand surgeons analyzed the findings using double-blinded X-ray and CBCT and categorized fractures as consolidated based on a 50 % visibility threshold concerning trabecular bridges. Proximal pole sclerosis, communition, cyst formation, and humpback deformity were similarly analyzed for all cases. Agreement between readers was calculated using Kappa, and sensitivity, specificity, and accuracy using RStudio software. The gold standard was the radiologic and clinical follow-up for all patients at two months.

**Results:**

Inter-reader agreement between the four readers was moderate concerning X-ray (0.543) but substantial concerning CBCT (0.641). It was almost perfect between seniors regarding CBCT (Kappa = 0.862). Sensitivity, specificity, and accuracy were 0.75–0.78, 0.4, and 0.61–0.64, respectively, for two readers regarding CBCT. The X-ray values were 0.65–0.71, 0.35–0.4, and 0.53–0.59, respectively.

**Conclusion:**

CBCT proves more accurate and reliable than X-ray for diagnosing scaphoid union at an early follow-up and prevents longer immobilization and interruption of activity or work.

## Highlights

• Scaphoid union is controversial regarding diagnosis of healing.
• XR is the most used modality with poor reliability and reproducibility.
• CBCT allows with higher confidence diagnosis of consolidation avoiding longer immobilisation.
• Evaluation for translation is better with CBCT than XR at un early follow up.
• Six weeks is a reasonable delay to perform the first imaging follows up in waist scaphoid fracture.

## 1 Introduction

Scaphoid fractures occur frequently in young, active patients [1]. The scaphoid is the most commonly fractured carpal bone, representing approximately 70 %–80 % of all carpal fractures [2,3] with an annual incidence of 1/10,000 emergencies [4]. Overall, 75 % of the scaphoid surface is covered by cartilage, and its blood supply mainly originates from the radial artery with a retrograde blood flow [5]. This specific configuration increases the risk of pseudarthrosis in fracture cases, as the blood flow to the proximal part is easily compromised [6,7]. Hence, if a fracture is poorly treated or even unrecognized beyond four weeks after the trauma, the risk of non-union can reach up to 45 % [8]. The consequences of pseudarthrosis can be disastrous, as it possibly leads to the predictable, gradual development of early osteoarthritis, called scaphoid non-union advanced collapse [9].

Clinically, for diagnosing a scaphoid fracture, there are three main signs to consider: a tender anatomic snuffbox, painful palpation of the scaphoid tubercle, and positive piston (corresponding to pain with the axial compression of the thumb). Even in the acute stage, these three signs display low specificity. Nevertheless, a retrospective study has demonstrated that a scaphoid compression test (piston), which is the most reliable test (sensitivity [Sn] 100 %/ specificity [Sp] 80 %) in the acute stage [10], tended to become negative when the fracture has healed [11]. Dias et al. revealed that, among the patients with non-union, most continue to experience pain and restricted wrist mobility [12]. However, this is not always the case because the correlation between pain and scaphoid consolidation is fair, as some patients with a radiological non-union will be pain-free [1,11]. Thus, the clinical diagnosis of scaphoid healing is almost impossible at 6–8 weeks’ follow-up based on a physical examination only [3]. Moreover, the long-term immobilization caused by a cast exerts a relevant impact on patients’ daily lives, their ability of work, and their socio-economic efficiency [1,13].

Typically, radiological examinations are routinely performed based on X-ray (XR). The protocol includes antero–posterior (AP), lateral (LAT), and Schreck positions. However, there is controversy in the literature about the time of imaging assessment and reliable radiological signs of scaphoid healing [14]. Thus, several teams encourage performing multidetector CT (MDCT) scans in order to confirm scaphoid healing [15]. MDCT enables an excellent analysis of cortical bone, but it involves radiation exposure [16]. Cone-beamed computed tomography (CBCT) is a new dedicated extremity imaging method; its main advantages include a high spatial resolution [17], which permits a detailed analysis of bone architecture; lower radiation exposure, and a smaller field of view (FOV) compared to MDCT [18,19].

This study sought to compare CBCT to XR in terms of scaphoid healing based on the expertise of junior and senior radiologists and hand surgeons, and to evaluate the accuracy of CBCT as an affective imaging modality at an early post-fracture stage.

## 2 Materials and methods

This prospective study involved patients who were diagnosed with a scaphoid fracture between April 2018 and March 2019, and its protocol was approved by the institutional ethical committee (CCR number 2017−01276).

### 2.1 Population

Overall, 52 patients with a scaphoid fracture who underwent XR and CBCT six weeks after injury were successively included in the study. The mean age was 34 (range: 13–77) years, and 43 patients were male (81 %). Fourteen patients were smokers, four were unemployed, 11 were students, 18 were manual workers, 16 were office workers, and three were retired. Ninety percent were right-handed (48). Twenty-six fractures were on the ride side, and 26 on the left side.

### 2.2 Physical examination

At the initial physical examination, all patients presented painful scaphoid tubercle palpation and a positive piston sign. Patients were seen in the emergency department, and they all received XR the day of consultation. Fractures were described according to Schernberg classification [20]. After the fracture diagnosis, all patients had a follow-up in the hand clinic. All scaphoid fractures were immobilized with a short arm cast without thumb. At the six-week follow-up all patients underwent XR and CBCT to document the consolidation. All patients were seen clinically around eight weeks post trauma.

### 2.3 Imaging analysis

Each patient underwent XR and CBCT the same day. CBCT (OnSight, Carestream Health, Rochester, New York) displays a gantry featuring a 58-cm patient aperture and movable table. The wrist was scanned with the patient in the sitting position, the arm extended, and the hand in the prone position. The acquisition parameters for the wrist are summarized in Table 1. Prior to the acquisition, two scouts were performed, consisting of one antero-posterior (AP) and one lateral (LAT). All A-CBCT images were reconstructed in coronal and sagittal planes with the bone kernel (window width: 1500; window level: 300). All images were reconstructed using first-generation model-based iterative reconstruction and metallic artifact reduction in cases of metallic device. XR (Siemens, ISIO and Philips, DigitalDiagnost) was performed in AP, LAT, and Schreck positions. The parameters applied for the XR are summarized in Table 1.

**Table 1:** Scanning parameters of the CBCT (OnSight, Carestream Health, Rochester, New York) and XR (Siemens, ISIO and Philips, DigitalDiagnost).

Parameters	CBCT	XR
Current	5mA	2.8mAs
Energy	80kVp	44kVp
FOV	216 × 216 mm	
Isotropic voxel size (m)	884 × 884	
Rotation time	25.18 s	
Exposure time	21 s	
Scanning slice thickness	0.26mm	
Scan rotation angle	216.5°	
Focus-detector distance		120 cm

CBCT: cone-beam computed tomography; XR: X-ray; s: second; dose index.

All images were anonymized, classified randomly for XR and CBCT, and analyzed separately in different sessions by four readers. Analysis was performed in Osirix session (Osirix^R^, Pixmeo SARL, Bernex, Switzerland).

### 2.4 Quantitative analysis

The independent observers were two musculoskeletal radiologists (a junior with seven years of experience and a senior with 16 years of experience) and two hand surgeons (a junior with five years of experience and a senior with nine years of experience). The four readers analyzed XR and CBCT in a double-blinded manner and categorized the fractures as consolidated according to a 50 % visibility threshold concerning trabecular bridges. The gold standard was the radiologic and clinical follow-up for all patients at two months (next clinical and radiographic follow-up).

### 2.5 Qualitative analysis

The presence of proximal pole sclerosis, communition, cyst formation, and humpback deformity were similarly analyzed for all cases by all readers.

### 2.6 Statistical analysis

For scaphoid consolidation, we first evaluated the agreement between readers using Kappa (between two readers) and Kappa Fleiss (among more than two readers) for XR and CBCT, respectively. Values ≤0 were considered to indicate no agreement, 0.01–0.20 none to slight, 0.21–0.40 fair, 0.41–0.60 moderate, 0.61–0.80 substantial, and 0.81–1.00 almost perfect agreement. Then, the accuracy of XR and CBCT was assessed through a clinical and radiographic follow-up at two months. Sensitivity, specificity, the positive predictive value (PPV), and the negative predictive value (NPV) were calculated. We used RStudio software (R 3.5.1) for these assessments. Afterwards, we compared XR and CBCT in terms of scaphoid proximal pole density, the presence of translation, the presence of humpback deformity, communition, and cyst formation, and all criteria together for the best treatment choice. After testing the data’s lognormality by considering a p-value of ≤0.05 value to signify statistical significance (alpha), we used the Wilcoxon test and Spearman’s rank correlation with a 95 % confidence interval. GraphPad Prism 8 (San Diego, California) was employed for these statistical analyses.

## 3 Results

Fractures were localized in 44 % cases in Schernberg’s zone IV, 33 % in zones II–III, and 23 % in zones V–VI. The difference between the two modalities of analysis was non-significant (p = 0.572). The correlation test revealed an r-value of 0.569 with p < 0.0001.

**Fig. 1:**
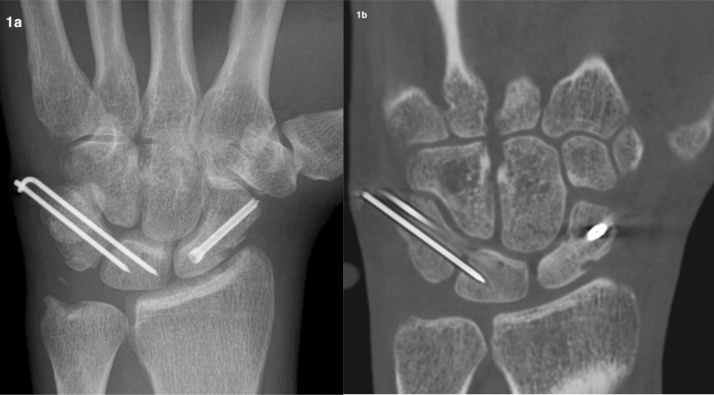
Manual worker aged 34 years with an unstable fracture in Schoenberg’s zone III of the right scaphoid according to Mayo criteria [21] treated with surgery to stabilize the fracture. XR (a) showed no healing, according to all readers. To the contrary, CBCT (b) showed total agreement of scaphoid consolidation.

The mean immobilization duration with a short arm cast without thumb was seven (range: 4–12) weeks. Twelve patients had an unstable fracture, according to Mayo criteria [21]; they underwent surgery to stabilize the fracture (Fig. 1).

At clinical follow-up at two months, 79 % (41) patients had no pain, and 21 % (11) presented with pain. Of the 11 patients with pain, five had an associated fracture (one complex distal radius, one hamatum fracture, one radial head fracture, and two trans-scapho retro-lunar dislocations); two had a tenosynovitis (one de Quervain and one flexor carpi radialis synovitis); one presented with a complex regional pain syndrome. Moreover, three patients with pain had a previous scaphoid pseudarthrosis that was identified during this new trauma and treated by means of surgery.

**Fig. 2:**
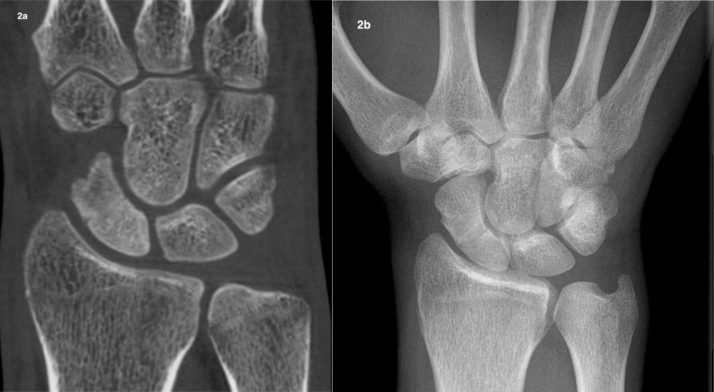
CBCT (a) of a 36-year-old, office worker with a right scaphoid fracture in Schoenberg’s zone III with complete healing according to all readers. XR (b) of the same patient shows no evidence of scaphoid healing.

In terms of scaphoid consolidation, according to the four readers, agreement was moderate for XR (0.543) and substantial for CBCT (0.641). According to the radiologists, agreement for XR was fair (0.35) and almost perfect according to the hand surgeons (0.956). However, agreement was substantial among hand surgeons and radiologists (0.803) for CBCT. The agreement for XR was slight among juniors (0.176) and fair among seniors (0.354). Regarding CBCT, agreement was superior among juniors though still moderate (0.441) and almost perfect among seniors (0.862). Regarding the accuracy of CBCT (Fig. 2a), the value was 0.63 for the senior radiologists (Sn, 0.78; Sp, 0.4; PPV, 0.67; NPV, 0.53) and 0.61 for the senior hand surgeons (Sn, 0.75; Sp, 0.4; PPV, 0.66; NPV, 0.5). The accuracy of XR (Fig. 2b) was 0.53 for the senior radiologist (Sn, 0.65; Sp, 0.35; PPV, 0.61; NPV, 0.38) and 0.59 for the senior hand surgeon (Sn, 0.71; Sp, 0.4; PPV, 0.65; NPV, 0.47) (Table 2). With reference to scaphoid proximal pole density, the presence of humpback deformity, communition, and cyst formation, no significant differences were observed (p < 0.125, 0.120, 0.75, and 0.343, respectively). Furthermore, no significant correlation was indicated based on the r-values (0.214, –0.05, 0.179, and –0.09, respectively) for the same criteria. A significant difference was only detected for translation (p < 0.0002), along with a significant correlation (r = 0.290) (Table 3).

**Table 2:** Comparison of statistical analysis in terms of CBCT and XR for scaphoid consolidation.

	XR	CBCT
Agreement among four readers	0.543	0.641
Agreement among hand surgeons	0.95	0.803
Agreement among radiologists	0.35	0.803
Sensitivity	0.75	0.78
Specificity	0.4	0.4
VPN	0.5	0.53
VPP	0.66	0.67
Accuracy	0.53- 059	0.61−0.63

CBCT: cone-beam computed tomography; XR: X-ray; VPN: negative predictive value; VPP: positive predictive value.

**Table 3:** Comparison of qualitative analyses between CBCT and XR.

	P (Wilcoxon test)	Correlation (Spearmann r)
Schenberg zone	0.5722	0.5698
Bridges	0.6072	0.3397
Overall impression	0.7744	0.4783
Density	0.125	0.2141
Translation	0.0002	0.2906
Humpback deformity	0.2101	−0.05495
Communition	0.7539	0.1797
Cyst formation	0.3438	−0.09759

CBCT: cone-beam computed tomography; XR: X-ray.

## 4 Discussion

Our study highlights the superior accuracy of CBCT compared to XR for predicting scaphoid healing at early follow-up (six weeks), thus enabling a faster mobilization of patients and return to daily activity and work. This study demonstrated the relevance of the doctor’s experience, regardless of speciality, when analyzing scaphoid consolidation when we compared senior and junior physicians, with an improved result when using CBCT.

Until now, XR has been a well-known follow-up method designed to detect bone fracture consolidation [22].

The formation of trabecular bridges and cortical fusion are accepted signs of bone healing [23]. However, the amount of trabecular and bone formation reported to whole the bone and the exact moment when a radiological follow-up should be conducted remain controversial [22]. It is known that XR displays a poor inter-observer agreement, which is not improved by clinical experience concerning scaphoid fracture detection [24]. In the early post-trauma period, Dias et al. demonstrated that XR cannot be used to identify scaphoid consolidation due to the difficulty of identifying the trabecular crossing of the fracture and sclerosis of the fracture line in the context of the shape and orientation of the scaphoid bone [25,26]. Hannemann suggested that XR at six weeks after injury can be reliably used so as to determine whether scaphoid waist fractures (corresponding to Schernberg’s zones II, III, and IV) have actually healed (k = 0.816 for non-union) [27]. However, when documenting the union (partially or complete) based on conventional radiographic imaging displays less inter-observer agreement (k = 0.390).

In this context, instead of XR, the CT scan was investigated and shown to document scaphoid union. This technology enables better spatial resolution and permits the image reconstruction in the scaphoid longitudinal axis. Buijze reported that the inter-observer reliability of the CT scan to document scaphoid waist union using any degree of bony bridging proved to be substantial (k = 0.660); Sn was 78 %, Sp was 96 %, and accuracy was 84 % [28].

We already demonstrated that CBCT displays a higher Sn and Sp for detecting scaphoid fracture [29]. This technique is also used to detect occult fractures of the wrist and traumatic diseases [30,31]. To the best of our knowledge, there are presently no data available concerning both the use and validation of CBCT for scaphoid consolidation. This technique is emerging for diagnosing diseases of the extremities. Several studies have reported on the use of this technique for the early diagnosis of fractures, including the analysis of lower extremities in an upright position, the detection of syndesmosis injuries while using different angles for visualizing the lower extremities. This technology is also often used in dentistry in pediatric populations [[32], [33], [34], [35]]. In addition, this technique enables a lower radiation dose compared to CT (comparison between dose on XR and CBCT) [36]. In our study, concerning the consolidation, the interobserver agreement among juniors was moderate (k = 0.441) but almost perfect among seniors (k = 0.862). This result confirms the relevance expertise for detecting scaphoid healing. Buijze demonstrated that training improves interobserver reliability for the diagnosis of scaphoid fracture displacement [37]. The average Sn, Sp, and accuracy of diagnosing scaphoid fracture displacement were 83 %, 85 %, and 84 % for the nontraining group versus 87 %, 86 %, and 87 % for the training group. There are no available data in the literature concerning the effect of experience or training on interobserver agreement with respect to fracture healing. Among the four readers, the interobserver agreement was moderate for XR (k = 0.543) and substantial for CBCT (k = 0.641), which indicates that CBCT is a more reproducible technique than XR for diagnosing scaphoid union at an early follow-up.

Concerning fracture displacement, it is known that XR des not sufficiently detect scaphoid displacement [38]. Based on our results, we conclude that translation assessment was superior with CBCT than XR, with a significant correlation (r = 0.290; p < 0.0002). We confirmed what Lozano-Calderon already demonstrated as regards CT: the interobserver reliability of CT (k = 0.43) and CT and XR (k = 0.48) improved compared to using XR alone (k = 0.27) for determining scaphoid displacement [39]. Even if there were no significant correlation for humpback deformity, scaphoid proximal pole density, or comminution, we strongly believe that, in addition to diagnosing scaphoid union, CBCT can accurately evaluate scaphoid displacement. This may be accounted for by that the majority of our study subjects had a stable fracture, and only 12 patients underwent surgery (23 %). Meanwhile, in the literature, the rate of unstable fracture is estimated at >50 % [40]. In addition, fractures that are predominately in zone IV and the distal pole of the scaphoid may explain the low rate of proximal pole scaphoid sclerosis.

Concerning the duration of immobilization due to a cast, the standard care is still 8–12 weeks in the literature, which is, however, mainly based on XR follow-up [41,42]. The duration depends primarily on fracture localization because a vascularization distal pole fracture can heal within four weeks, whereas a proximal pole can take up to 12 weeks to heal without surgery, depending on whether the patient is a smoker or not [14]. As there is no reliable clinical sign of consolidation, surgeons use images to take decisions about discontinuing immobilization. It is known that waist fractures represent two-thirds of all scaphoid fractures. In this context our study is in agreement with the published literature, with 77 % of the fractures in the waist zone (Schernberg’s zones II, III, and IV) [43]. Overall, 90 % of waist fractures have been reported to be healed after six weeks of cast immobilization with a CT follow-up. This is the exact reason why we chose this timeline, based on the trauma and CBCT, to document consolidation [44,45]. In our study, at the six-week follow-ups, 70 % of fractures were considered healed with according to CBCT [46]. We noticed that patients who needed longer immobilization were often younger patients, or those with associated fractures (one complex distal radius, one hamatum fracture, one radial head fracture, and two trans-scapho retro-lunar dislocations), or with an undiagnosed pseudarthrosis that was discovered at trauma diagnosis. We have confirmed herein that the high percentage of scaphoid waist fractures heal after six weeks, and CBCT enables a shorter immobilization period. The mean immobilization time was seven weeks due to the delay between CBCT and hand surgeon consultation. Fifty-two percent of patients were office workers, and we know that, in this population with non- or minimally physical demands, that a short arm cast immobilization allows for the majority of patients to return to work fairly soon. However, for manual workers (35 % of our subjects), returning to work requires the healing of the scaphoid fracture as determined by CT (>50 % of trabeculae bridging across the fracture site) [14]. However, beyond the radiological aspect, these manual workers need a range of motion and grip strength amounting to 20 %–40 % of the contralateral side to return to work [14]. We observed this phenomenon in our patients—for office workers, the mean time after which they could return to work was four weeks, and for manual workers, it was 16 weeks. In this context, CBCT plays an essential role in investigating consolidation six weeks after the trauma, as well as in the re-education process for manual workers so that they can return to work as soon as possible.

Our study displays several limitations. The small size of the population is due to the fact that we only included patients who underwent both XR and CBCT on the same day at six weeks and then immediately attended a consultation with a hand surgeon to confirm scaphoid healing. Complications such as humpback deformity and proximal pole sclerosis were rarely seen in this study, which can be explained by the stability of fractures and involvement of waist zones, respectively. Another limitation was that radiation doses in XR and CBCT were not compared. The radiation dose in CBCT is calculated based on the absorbed dose rather than the effective dose. A separate supplementary phantomic study should be conducted to estimate the effective radiation dose of CBCT for wrists. Finally, this study did not compare CBCT and MDCT as the latter is not recommended for scaphoid healing follow-up, though is only used in complicated cases with poor progression.

### 4.1 Conclusion

Although the moment of follow-up regarding scaphoid healing remains a controversial topic, CBCT could shorten this period enabling the diagnosis of consolidation with higher confidence than XR. CBCT technology provides more reproducible results than XR regardless of the experience of the radiologist or hand surgeon. Finally, the lower radiation dose permits the technique’s repetition for following-up scaphoid healing. Six weeks appears to be a reasonable period after which the first imaging follow-ups should be performed.

## Disclosure paragraph

1) The scientific guarantor of this publication is Dr Sana Boudabbous.

2) The authors of this manuscript declare no relationships with any companies, whose products or services may be related to the subject matter of the article.

3) The authors state that this work has not received any funding.

4) No complicated statistical analyses were performed in this study

5) Institutional Review Board approval was obtained.

6) Written informed consent was waived by the Institutional Review Board.

7) Only if the study is on animals: not applicable

8) Some study subjects or cohorts have been previously reported: no previous publication of this study.

9) Methodology:

• Prospective

• Diagnostic

• Performed at one institution
